# The dual role of silver during silicon etching in HF solution

**DOI:** 10.1186/1556-276X-7-455

**Published:** 2012-08-13

**Authors:** Manel Abouda-Lachiheb, Nesma Nafie, Mongi Bouaicha

**Affiliations:** 1 Laboratoire de Photovoltaique, Centre de Recherches et des Technologies de l’Energie, Technopole de Borj-Cedria, BP 95, Hammam-Lif, Tunis, 2050, Tunisia

**Keywords:** Silicon nanowires, Silver, HF, Etching, Catalyzing

## Abstract

It was reported that during silicon etching, silver was subjected to have a controversial role. Some researchers debate that silver protects silicon, and, at the same time, other ones confirm that silver catalyzes silicon underneath. In this paper, we give experimental results arguing the dual role that silver has during the formation of silicon nanostructures. We give a proof that the role of silver depends on the experimental details and the intrinsic properties of silver during its deposition on the silicon wafer. Through our investigations, we tracked the silver particles that indicated which mechanism is involved. Characterizations of the prepared samples were made using a scanning electron microscope.

## Background

By etching silicon in a HF aqueous solution, porous silicon can be formed. When silicon etching in the HF solution is assisted by silver, well-organized nanostructures like silicon nanowires [1-3] are formed. The latter are very interesting in electronic and photovoltaic application. In order to enable significant improvements in the devices’ technologies, understanding of the fabrication mechanism must be attained to produce materials with precise control. Herein, we are interested in the silver-assisted chemical etching of silicon in HF solution. This technique can be realized either by a one-step process or a two-step process. The one-step method includes, at the same time, metal deposition and electroless etching. In this process, the silicon substrate is immersed in an AgNO_3_/HF aqueous solution. The two-step method consists first of metal deposition and second, the chemical etching. In the latter method, the silver particles are evaporated or chemically formed on the surface of the silicon sample then introduced in a HF aqueous solution. With these two processes, we obtain two different behaviors of the silver particles; in one case, the silver catalyzes silicon, and in the other one, it protects the silicon underneath. Therefore, using a scanning electron microscopic (SEM) characterization, we tracked silver particles during silicon etching to determine which mechanism is privileged. To interpret the obtained results, we analyze charge displacement at the metal/Si interface. The developed model clarifies the role that the silver has during silicon metal-assisted etching experiences. Hence, the controversy regarding this role is solved.

## Methods

For cleaning, samples were boiled in acetone for 10 min, followed by their immersion in ethanol for 5 min to remove organic greases. Then, we rinsed them three times with deionized water. Finally, samples were dipped in a 5% aqueous HF solution for 1 min to eliminate native silicon dioxide. This study was realized at two ambient conditions: room temperature and atmospheric pressure. The etching duration was 1 h. We used high purity single-crystalline silicon. The wafers are p-type, boron-doped and (100)-oriented, with a thickness of 525 μm and a resistivity of 1 to 10 Ωcm. The samples were then separated in sample A and sample B to be treated differently. Sample A was immersed in an etching solution composed of HF/AgNO_3_[1-3]. The concentrations in the latter were 40% and 0.02 M, respectively. On sample B, we first evaporated a 30-nm silver film, and then it was immersed in a 40% HF aqueous solution [4].

Our SEM observations show two different structures; in the case of the one-step process (sample A), we obtained silicon nanowires (SiNWs) covered with a dendritic silver film. We did not observe any silver nanoparticle at the bottom of SiNWs. In the two-step process (sample B), we obtained silicon pores. However, Fang et al. [4] obtained SiNWs by etching a silicon wafer on which they evaporated a 20-nm silver film.

## Results and discussion

**Figure 1:**
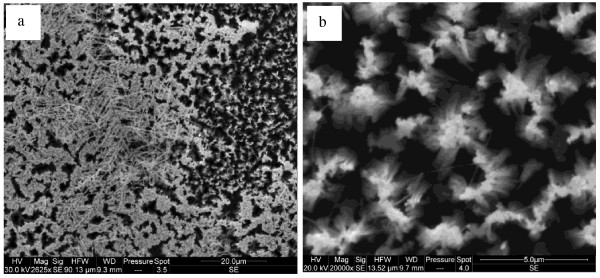
** SEM images of sample A.** (**a**) silver dendrites and nanoparticles and (**b**) magnification of silver nanoparticles.

In Figure 1, we give the top section SEM images of sample A, where the silver nanoparticles cover the top surface of the SiNWs. The silver film formed on wafer A is not uniform and not compact. The nonhomogeneity of this film can be attributed to the surface status. It was reported in several works [5-7] that the dendrites formation is related to the electrons’ exchange between silicon atoms and silver ions. In Figure 1a, we observe in some regions silver nanoparticles on top of SiNWs, where the image in Figure 1b is the magnification of this zone. In other regions, the silver forms a filament-like structure. The growth mechanism of silver dendrites is still unclear; the diffusion-limited aggregation theory [8] may play an important role to explain this mechanism. We notice that we did not see any silver nanoparticles at the bottom of SiNWs.

**Figure 2:**
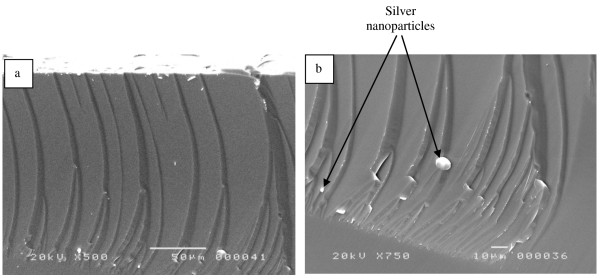
** SEM images of sample B.** (**a**) Tilted cross section and (**b**) a magnification showing the silver particles.

However, in the case of sample B, where a 30-nm silver film was deposited before the etching step, we observe pores formation at a depth of 200 μm as shown in Figure 2. Figure 2a is a tilted SEM image of the sample showing both the top surface and the cross section. We notice the beginning of pores from the top of the wafer and the presence of silver nanoparticles in the bottom of pores which is magnified in image (Figure 2b). The formed pores are not uniform. Hence, we observe the pores at different depths. The average of the pore diameters is around several micrometers. This could be due to the thickness of the silver film.

From the obtained results for samples A and B, we estimate the etching rate for sample A at about 0.5 μm/min and the penetration rate of silver particles for sample B at about 2.7 μm/min.

In this work, we aim to explain the mechanism governing silicon etching to obtain SiNWs or pores. In the two metal-assisted etching structures, the intrinsic properties of the metal orientate the silicon etching. The latter is initiated by the formation of silicon dioxide. It was reported [9] that silver-assisted etching of silicon, in presence of H_2_O_2_, leads to a homogenous and large film SiNWs. In addition, the etching reaction is 250 times lower in absence of H_2_O_2_[9]. However, as obtained in the present work and reported by Peng et al. [1], SiNWs could be also obtained without H_2_O_2_. In the used solution in this work, there are many oxidizing agents, and the most important one is the (NO_3_)^−^ ions. The principal reactions explaining the silicon dissolution are the following:

(1) $${\text{AgNO}}_{3}\to {\text{Ag}}^{+}+{\left({\text{NO}}_{3}\right)}^{-}$$

(2) $$\text{HF}\to {\text{F}}^{-}+{\text{H}}^{+}$$

(3) $${\text{H}}^{+}+{\left({\text{NO}}_{3}\right)}^{-}\to {\text{HNO}}_{3}$$

(4) $$3\;\text{Si}+{4\;\text{HNO}}_{3}\to {3\;\text{SiO}}_{2}+4\;\text{NO}+{2\;\text{H}}_{2}\text{O}$$

(5) $${\text{SiO}}_{2}+6\;\text{HF}\to {\text{H}}_{2}{\text{SiF}}_{6}+{2\;\text{H}}_{2}\text{O}$$

The electroless metal deposition mechanism was presented by Morinaga et al. with copper particles [10]. Then Peng et al. started with the protecting mechanism that silver has during the silicon etching in the one-step method [1]. After that, they provided in [11] their new position regarding the etching processes of silicon wafers in aqueous HF/AgNO_3_ solution where the catalyzing effect was described. Qiu et al. [12] gave experimental evidences that silver protects the silicon underneath. Consequently, the role that silver has was still not elucidated.

**Figure 3:**
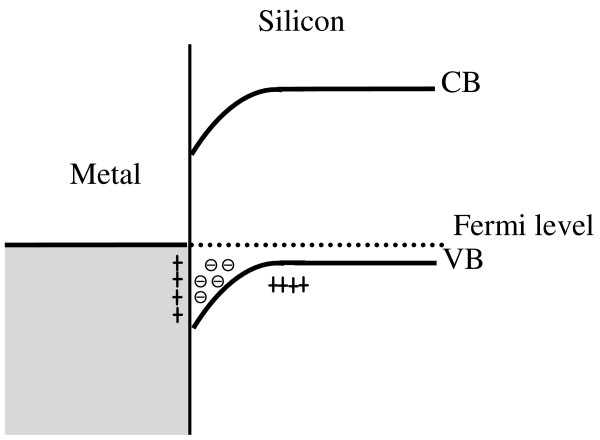
Schematic band diagram at the Ag/Si interface.

In this section, we give the explanation of SEM images in Figures 1 and 2. Therefore, to understand the etching process of samples A and B, we propose a unique interpretation of the experimental observations in both cases by analyzing what happens at the silver/silicon interface. In Figure 3, we give the schematic band structure at the silver/silicon interface. In such a case, the work function of silver (*ϕ*_Ag_ = 4.3 eV) is less than that one of silicon (*ϕ*_Si_ = 4.9 eV). Consequently, at contact, electrons diffuse from the metal to the semiconductor until the thermodynamic equilibrium is reached at the same Fermi level.

The scheme in Figure 3 explains the Ag/Si interface in the case of evaporation (sample B). However, using work functions of Ag and Si becomes impossible when the silver is at its ionic form. This is related to the fact that the work function of a metal is defined as being the needed energy to extract one electron from its metallic conductor. Unfortunately, this is not applicable in the case of silver ions when they capture electrons from silicon. In such a case, we have to use electronegativity which is the aptitude of an element to attract electrons. In Table 1, we give values of the electronegativity of Ag, Si, O, and B (boron) elements involved in the etching mechanism. The electronegativity of the silver ion is greater than that of silicon. The electrons move from silicon to silver ions. At contact, the silicon underneath the silver particles becomes more positively doped than the silicon around silver.

**Table 1:** **Values of electronegativity of Ag, Si, O, and B**[13]

**Elements**	**Electronegativity**
Ag	1.93
Si	1.90
O	3.44
B	2.04

**Figure 4:**
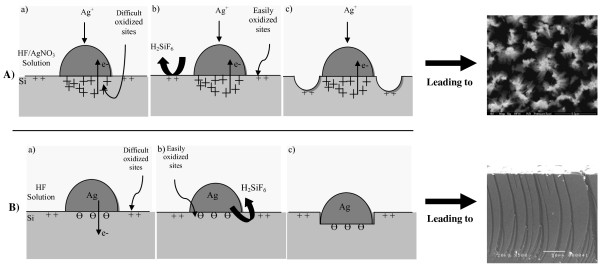
** Schematic illustration of the etching mechanism.** (**A**) the one-step process and (**B**) the two-step process.

The aptitude of oxidizing silicon depends on the silicon status; from values in Table 1, we can deduce that oxidizing pure silicon substrate (intrinsic silicon) is easier than oxidizing pure boron material. We can extrapolate that boron-doped silicon has an effective electronegativity value ranging between 1.90 (Pure Si) and 2.04 (Pure B). Consequently, in the case of sample A where silver ions are deposited on the Si substrate, there is transfer of electrons from Si to silver. Therefore, the region underneath silver becomes poor in electrons as compared to the region around silver nanoparticles. Hence, oxidizing Si around silver particles (P-doped Si) becomes more important than the Si underneath it, which behaves like P^++^-doped Si. Thus, the etching of oxidized sites around silver particles starts leading to the formation of SiNWs (Figure 4A, processes a-c). However, in the case of sample B, due to the neutral region formed underneath the evaporated silver nanoparticles, Si around them (P-doped Si) is a difficult oxidized region as compared to Si underneath them that behaves like intrinsic Si. As a result, etching starts underneath Ag particles leading to pores formation (Figure 4B, processes a-c).

**Figure 5:**
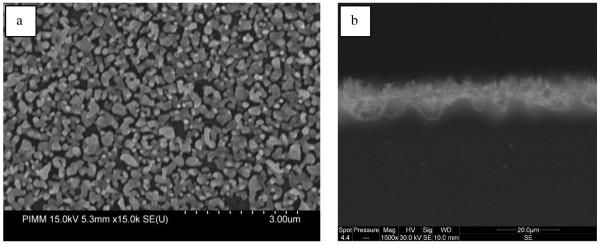
Top and cross-section SEM images of sample C (a) chemically deposited Ag film and (b) after removing the Ag film.

**Figure 6:**
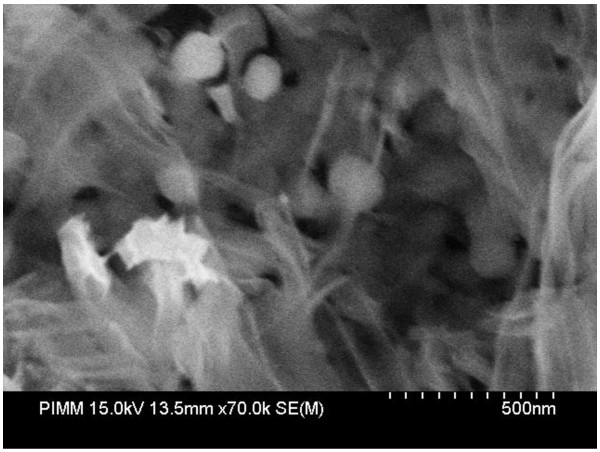
Top SEM image of sample C after etching in HF solution.

To confirm the above mechanisms, we use twin samples (sample C). After cleaning, samples were immersed for a short duration (3 min) in a HF/AgNO_3_ solution with the same concentrations used for sample A. In Figure 5, we show a top SEM image of one sample where we observe silver nanoparticles deposited in the HF/AgNO_3_ (image a). However, image b is a cross-section SEM view of the second sample after removing the silver film, where we observe the beginning of silicon etching. Then, the sample C with silver nanoparticles was immersed in a 40% HF aqueous solution, as done for sample B, for 2 min. Figure 6 is a SEM image of this sample after etching, where we observe silver nanoparticles in the bottom of etched silicon.

**Figure 7:**
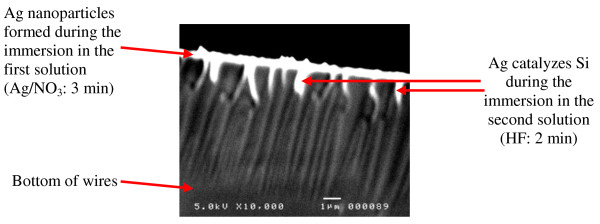
Cross-section SEM image of sample C showing both protecting and catalyzing effect of Ag nanoparticles.

According to our assumptions on the role of silver during the silicon etching, the one-step process starts during the 3 min in the HF/AgNO_3_ solution. The two-step process starts when the sample is immersed in sole aqueous HF solution, since silver nanoparticles are deposited in the HF/AgNO_3_ solution. In Figure 7, we give the cross-section SEM image of sample C (with silver nanoparticles) where we can see that no silver nanoparticles are found in the bottom of pores. However, the Ag nanoparticles are found on the top (white color) which confirms the one-step process. In addition, it is clear in our observation that silver nanoparticles start their catalyzing effect (the two-step mechanism) by digging silicon at depths of 1–2 μm.

## Conclusions

During metal-assisted etching experiences, some researchers reported that silver protects silicon. However, others confirmed that silver catalyzes the silicon underneath. In this paper, we give experimental results arguing the dual role that silver has during silicon etching. We propose a model based on the effect of electron transfer at the silver/silicon interface when silver is deposited during the HF etching (one-step method) and when silver is deposited prior to the etching in a HF solution (two-step method). By the proposed model, it becomes clear how silver nanoparticles can protect the silicon underneath in some experimental conditions and how they initiate pore formation by etching silicon underneath in others.

## Competing interests

The authors declare that they have no competing interests.

## Authors’ contributions

MAL prepared samples, performed the SEM imaging, and proposed the mechanisms of silicon etching. She also wrote the manuscript. NN helped on the SEM investigations. MB supervised the work, improved the proposed mechanisms and the text. All authors read and approved the final manuscript.
